# The Influence of Meal Frequency and Timing on Health in Humans: The Role of Fasting

**DOI:** 10.3390/nu11040719

**Published:** 2019-03-28

**Authors:** Antonio Paoli, Grant Tinsley, Antonino Bianco, Tatiana Moro

**Affiliations:** 1 Department of Biomedical Sciences, University of Padova, 35131 Padova, Italy; 2 Faculty of Sport Sciences, UCAM, Catholic University of Murcia, 30107 Murcia, Spain; 3 Department of Kinesiology & Sport Management, Texas Tech University, Lubbock, TX 79409, USA; grant.tinsley@ttu.edu; 4 Department of Psychology, Educational Science and Human Movement, Sport and Exercise Sciences Research Unit, University of Palermo, 90144 Palermo, Italy; antonino.bianco@unipa.it; 5 Department of Nutrition and Metabolism, University of Texas Medical Branch, Galveston, TX 77550, USA; tamoro@utmb.edu; 6 Sealy Center on Aging, University of Texas Medical Branch, Galveston, TX 77550, USA

**Keywords:** time-restricted feeding, fasting, meal frequency, meal timing, obesity, cardiovascular health, diabetes

## Abstract

The influence of meal frequency and timing on health and disease has been a topic of interest for many years. While epidemiological evidence indicates an association between higher meal frequencies and lower disease risk, experimental trials have shown conflicting results. Furthermore, recent prospective research has demonstrated a significant increase in disease risk with a high meal frequency (≥6 meals/day) as compared to a low meal frequency (1–2 meals/day). Apart from meal frequency and timing we also have to consider breakfast consumption and the distribution of daily energy intake, caloric restriction, and night-time eating. A central role in this complex scenario is played by the fasting period length between two meals. The physiological underpinning of these interconnected variables may be through internal circadian clocks, and food consumption that is asynchronous with natural circadian rhythms may exert adverse health effects and increase disease risk. Additionally, alterations in meal frequency and meal timing have the potential to influence energy and macronutrient intake.A regular meal pattern including breakfast consumption, consuming a higher proportion of energy early in the day, reduced meal frequency (i.e., 2–3 meals/day), and regular fasting periods may provide physiological benefits such as reduced inflammation, improved circadian rhythmicity, increased autophagy and stress resistance, and modulation of the gut microbiota

*“Eat like a king in the morning, a prince at noon, and a peasant at dinner”*

*(Moses ben Maimon or Maimonides. 1135-1404)*

## 1. A Brief Historical Introduction

In Western culture, it is a common idea that the daily food intake should be divided into three square meals: breakfast, lunch, and dinner. Often dieticians suggest adding two snacks (morning and afternoon) to help appetite control, and indeed the mainstream media message is to eat “five to six times a day”. However, the number of meals is not a universal standard, and the traditional three square meals are, somewhat surprisingly, a recent behaviour. As an example, the Ancient Romans had only one substantial meal, usually consumed at around 16:00 h (*coena*), and they believed that eating more than once per day was unhealthy. Although they also ate in the morning (*ientaculum*) and at noon (*prandium*), these meals were frugal, light and quick [1]. Later, Monastic rules influenced common peoples’ eating behaviour. The term breakfast means “break the night’s fast”, pointing out that it is the first meal after the evening/night devoted to prayer [2]. In the early medieval times, monks were obliged to remain silent during meals while one of them read aloud a religious text. One of the most-read texts was the *Collationes* (compilation) by Giovanni Cassiano, and it is worth mentioning that the Italian term for breakfast is *“colazione*”, which is derived precisely from the Latin word “*collationes*” [3]. Breakfast also became important during the industrial revolution as a meal consumed before going to work. Dinner in its current form and timing became popular after the widespread use of artificial light, which facilitated eating before dawn and after dark [3]. 

## 2. Meal Frequency

### 2.1. Epidemiological Studies about the Effects of Meal Frequency on Cholesterol, Body Weight and Diabetes

The origin of the firm belief that eating three meals per day is the better healthy choice is a mix of cultural heritage [4,5,6] and early epidemiological studies [7]. The available epidemiological studies have not primarily investigated cardiovascular diseases (CVDs), but rather some risks factors such as cholesterol and body weight [8,9]. These studies observed a worsening of blood lipids associated with a “gorging” (a reduced meal frequency, one or two meals daily) diet compared to “nibbling” (the consumption of frequent smaller meals or snacks). In these early studies, authors stated that a reduced meal frequency is associated with an increased risk of cardiovascular disease [10]. Subsequent studies seemed to confirm these previous findings, reporting a lower age-adjusted total and LDL (low-density lipoprotein) cholesterol in subjects who reported eating four or more meals daily, compared to those reporting one or two [11]. The association was also confirmed after adjustment for alcohol, smoking, systolic blood pressure, anthropometric measurements as WHR (waist to hip ratio) and BMI (body mass index), and macronutrient intake. In a 1989 paper, authors compared a very high frequency of meals (17) to a lower frequency (3) and found an improvement of total and LDL cholesterol with the higher frequency; however, this particular approach is clearly atypical in ordinary life [12]. A recent study within the European Prospective Investigation into Cancer (EPIC) project showed a lower concentration of total and LDL cholesterol in subjects reporting a higher (≥6 times/day) meal frequency compared to those who ate 1 or 2 times a day, even when adjusted for age, BMI, physical activity, smoking, total energy intake, and macronutrient distribution [13]. Again, a recently published cross-sectional analysis within the prospective Seasonal Variation of Blood Cholesterol Study in Worcester County, Massachusetts (SEASONS) showed that a frequency higher than four times per day leads to a lower risk of obesity compared to a frequency lower than three times per day, even after adjustment for age, sex, physical activity, and total energy intake [14].

Another large cohort study, the Malmo Diet and Cancer study, reported that eating more than six meals per day reduces the risk of obesity compared to less than three meals daily; moreover, after adjustment for diet and lifestyle, frequent eaters had lower waist circumference [15]. Regarding diabetes, a 16-year follow-up study showed an increased risk of type 2 diabetes mellitus in men who ate 1–2 times a day compared to those who ate three meals a day (relative risk RR 1.26) after adjustments for age, BMI, and other relevant factors [16]. These data are in contrast to another study that found no correlation between increased meal frequency and type 2 diabetes risk in women after six years follow up (3 times a day: RR 1.09, ≥6 times a day: RR 0.99) [17]. Despite the numerous studies examining risk factors, only one prospective cohort study investigated the relationship between meal frequency and coronary heart disease (CHD) risk. Cahill et al. [18] found that men eating 1–2 meals per day hadan RR for CHD of 1.10, men eating 4–5 meals per day hadan RR of 1.05, and men eating ≥6 times hadan RR 1.26, as compared to who ate three times a day after adjustment for total energy intake, diet composition, and other risk factors. In general, conflicting results are depending on the outcome investigated and the methodology used. 

However, as also suggested by other authors [19,20], the correlation between a reduced meal frequency and a higher risk of CHD in these studies appears to be weak considering the cross-sectional nature of these studies, making it difficult to establish the causality or temporality of this association.

### 2.2. Meal Frequency and Weight Control: One, Two, Three, or More Meals?

Obesity is a rapidly growing epidemic worldwide; its prevalence has nearly doubled in more than 70 countries since 1980. In 2015, a total of 107.7 million children and 603.7 million adults were obese [21]. Seventy-five percent of the world’s population live in countries where overweight and obesity kills more people than underweight [22]. Obesity is one of the main risk factors for cardiovascular disease, along with dyslipidemia and hypertension [23]. As a part of the strategies proposed for reducing energy intake (diets, drugs, and bariatric surgery) [24] and for increasing energy output (exercise and non-exercise movement) [25], meal timing and frequency could exert a significant influence on weight control and weight loss. [26,27]

A very recent and extensive study published by Kahleova and colleagues [28] investigated 50,660 adult members of Seventh-day Adventist churches in the United States and Canada. The results showed that eating one or two meals daily was associated with a relatively lower BMI compared with three meals daily. Interestingly, they found a positive relationship between the number of meals and snacks (more than three daily) and increases in BMI. Furthermore, the change in BMI was related to the length of the overnight fast: the longer the overnight fast, the lower the BMI. Authors suggested that the positive effects of such nutritional regimen are due to the combination of timing, meal frequency, and long overnight fasting; they hypothesised different underlying reasons as an effect of satiety hormones (leptin or ghrelin), an improvement of peripheral circadian clock (and therefore an improvement of key metabolic regulators such as cAMP response element-binding protein), and a reduction of oxidative damage together with a higher stress resistance [28]. These data suggest that 1–2 meals are better than three or more, but how can we integrate these results with previous, older research? Both older studies [9,10,12,29,30] and more recent research [31] seem to suggest that a higher meal frequency can reduce weight gain risk; however, recent large prospective studies seem to support that frequent snacking increases the risk of weight gain [32,33] and type 2 diabetes [16,17]. Additionally, research investigating acute metabolic responses to differing meal frequencies may support the benefits of a lower meal frequency. Taylor and Garrow evaluated the effects of isocaloric diets consisting of two or six meals per day on energy expenditure measured in a metabolic chamber. The results showed no differences during the day whilst night expenditure was significantly higher with two meals compared with six meals [34]. On the contrary, other studies demonstrated a significantly higher basal energy expenditure in the morning compared to the evening [35,36,37]. However, diurnal differences in the total energy expenditure are not consistently found in all studies [38]. Other studies suggest that weight gain and its metabolic consequences with a higher meal frequency are due to not only to the higher sugar derived energy intake [39] and associated metabolic issues, but also to increased food stimuli, hunger and desire to eat [40,41]. Thus, a regular meals pattern has potential positive effects on health outcomes regardless of meal frequency.

Often infrequent meal pattern, i.e. a reduced meal frequency, is associated with an irregular eating approach that could cause weight gain, increase hunger-related hormones, and ultimately lead to a metabolic disturbance that may increase cardiovascular risk [42]. On the contrary, a lower frequency but with regular timing may decrease weight gain risk [28]. 

### 2.3. Intervention Studies and Reciprocal Influences of Meal Frequency and Macronutrients

In addition to the effects of changing meal frequency per se, it must be considered that these changes could also modify the overall macronutrient intake. This was demonstrated by McGrath and Gibney, who convinced subjects who usually eat six times daily to reduce their frequency while persuading lower frequency eaters (three times daily) to increase their frequency to six times. The increase of meal frequency induced a significant reduction of total and LDL cholesterol but was coupled with a reduction of carbohydrate intake [30].

The reductions of cholesterol observed by McGrath and Gibney can be considered in light of the current debate about the real relationship between traditional disease markers such as total cholesterol and LDL cholesterol and CHD [43], as some have challenged the common idea that higher blood levels of cholesterol increase stroke and other cardiovascular events [44]. It is reasonable to assume that the mechanisms involved in cholesterol reduction may be related to cholesterol synthesis mechanisms. We now know that insulin activates a key enzyme in cholesterol biosynthesis, hydroxymethylglutaryl-CoA (HMGCoA) reductase (the target for statins) [45]. Even though the discussion about the mechanisms underlying this control (AMP-activated protein kinase, increased rate of transcription, or insulin-induced genes) [46,47,48], exceeds the aims of this review, it appears consequential that an increase in blood glucose and, of consequence, of insulin will lead to increased endogenous cholesterol synthesis [49,50,51]. It was demonstrated that a higher meal frequency (nibbling) reduced insulin concentrations as compared to three meals daily [12], likely caused by a reduction in cholesterol synthesis [29]. 

Apart from insulin’s action, another effect of a high meal frequency could be the increased cholesterol removal (reverse cholesterol transport) in the postprandial phase after a meal containing fat [52] and the inhibitory effects of cholesterol and fats on HMGCoA reductase [53]. We cannot dismiss the effects of macronutrient composition on meal frequency, blood lipids, and insulin effects. An increase in the number of snacks can also increase the amount of dietary protein [54]. Data suggest that whilst there is no correlation between number of snacks and hunger [54], or at least not a positive one [55,56], there is a greater fullness-related response with higher protein intake [41,55]. Thus, when discussing meal frequency, it is essential to also consider, from an ecological perspective, that changing meal frequency could also change the percentage of energy from particular macronutrients during the day. Moreover, substituting carbohydrates/sugars with protein in the snacks could change the outcome of low or high-frequency meals studies [39]. Finally, it is important to underline that meal frequency alone could not explain its effects on health’s outcomes. The contrasting data about meal frequency and health could be explained by the fact that, often, a reduced number of meals reflects an incorrect distribution: skipping breakfast, light lunch, and a high-calorie dinner or a very low number of meals (i.e., 1–2) could lead to poor metabolic control [16]. Moreover, the meal frequency effects are strictly related to meal timing and macronutrients uptake. At the moment the available data about the effects of nibbling (small, frequent meals) compared to gorging (large, infrequent meals) on isoenergetic conditions [57] provide conflicting results, probably due to the above-mentioned confounding factors. 

## 3. Meal Timing

### 3.1. Epidemiological Data on Meal Timing: Breakfast or Not Breakfast, This Is The Question

When considering meal frequency and timing, which meals are maintained or removed is not a minor issue. Generally speaking, those who consistently eat breakfast have a lower risk of weight gain compared to those who skip breakfast; moreover, those eating their largest meal at lunch or dinner have a greater risk of an increased BMI [28]. Moreover, Cahill et al. in 2013 discovered an interesting association between coronary heart disease (CHD) risk and frequency of consuming breakfast. Authors reported data coming from 51,529 healthy males (monitored from 1992 up to 2008) and concluded that “*eating breakfast was associated with significantly lower CHD risk*” [18]. Both dinner and breakfast skipping increased 24-h energy expenditure, concomitant with a longer fasting period, but skipping breakfast may elicit higher postprandial insulin concentrations and increased fat oxidation, suggesting a metabolic inflexibility that may lead to low-grade inflammation status and impaired glucose homeostasis [58]. In general, available data suggest that if there are health-promoting effects of reducing meal frequency, there may be differential effects of skipping breakfast versus dinner (i.e., evening fasting before an overnight fast vs. an overnight fast followed by continued morning fasting). Moreover, it has been suggested that late eating is related to increased risk of obesity and CHD [59] and also that a “grazing” eating pattern is related to higher total energy intake and later night-time food consumption [60]. Finally, there is a consensus about the association between breakfast consumption and CHD. Cahill et al. [18] published a large prospective study from the Health Professionals Follow-up Study on 26,902 American men aged 45 to 82 years. They found that men who skipped breakfast had a 27% higher risk of CHD compared with men who regularly ate breakfast (RR 1.27; 95% confidence interval CI 1.06–1.53). Additionally, eating late at night led to a 55% higher CHD risk (RR 1.55; 95% CI 1.05–2.29) compared to an earlier dinner. 

### 3.2. Intervention Studies and Meal Timing: Inner Clock Mechanisms

Furthermore, Jakubowicz et al. [61] demonstrated that an isocaloric diet differing in the distribution of calories during the day (i.e., high calorie in the morning vs. high calorie in the evening) could influence weight loss, serum ghrelin, insulin resistance indices, and subjective appetite feeling in overweight/obese women. The results confirmed the positive effects of consuming more calories earlier in the day, including through breakfast consumption, and the correlation between meal timing and body weight. However, it should be noted that some evidence has failed to support the importance of breakfast consumption for body weight change in free-living adults. Dhurandhar et al. [62] conducted a randomized controlled trial that assigned 309 overweight and obese adults to either eat breakfast or skip breakfast for 16 weeks. Despite high compliance with the assigned programs, they found that breakfast consumption did not produce weight loss relative to breakfast skipping. On the contrary, regarding cardiovascular health, Uzhova and collaborators found that skipping breakfast was associated with an increased risk of non-coronary and generalized atherosclerosis independent of conventional CVD risk factors in a sample of middle-aged asymptomatic individuals [63]. Moreover, Betts and colleagues showed that both lean and obese adults expend less energy during the morning when remaining in the fasted state than after consuming breakfast [64,65,66].

The opposite (i.e., the negative effects of late dining) is not so conclusive. Even though a recent meta-analysis demonstrated an association between evening energy consumption and higher BMI, they concluded that because of high heterogeneity it is difficult to draw conclusions about the effect of large evening dinner on weight control [67].

An important consideration related to early versus late feeding is the influence of feeding on the internal circadian clock [68,69,70,71]. The body circadian timing system is composed by a central clock in the hypothalamic suprachiasmatic nucleus and by different peripheral tissue clocks. The circadian clock system is involved in many metabolic rhythms including glucose and lipids. Whilst central clock dictates food intake, energy expenditure and insulin sensitivity, peripheral/tissues clocks carry out an additional control. For instance, the peripheral clock in the gut regulates glucose absorption and peripheral clocks in the adipose tissue and liver regulate their insulin tissue sensitivity while another peripheral clock in the pancreas regulates insulin secretion. Also lipids biosynthesis and catabolism are regulated in different tissue by a local molecular clock as demonstrated by recent studies on metabolomics and lipidomics.

It is well-known that disruption of central and or peripheral circadian clocks could promote obesity and CHD in many organisms [72]. Almost all species have developed an internal cellular clock mechanism, sensitive to the light and dark phases of a day, which allows animals to anticipate and to adapt to the changes in the environmental conditions linked to light and darkness. Early research performed in the 1970s identified the suprachiasmatic nucleus (SCN) as the main biological clock. The SCN regulates not only sleep-wake cycles but also many other physiological variables such as body temperature, blood pressure, hormone secretion, and behavioural variables. These circadian rhythms allow the organism to adapt to the environment and to be prepared for the different demands of daily life. For example, the morning increase of cortisol prepares the cardiovascular system for the upcoming day’s activities, and thus disruption of the circadian cortisol rhythm and the consequent cardiovascular impairment could lead to an increased risk of cardiovascular events in the early morning [73]. Another important marker of the internal clock is melatonin. Melatonin is strongly regulated by light/dark cycle with high levels during the night in all vertebrates. This fundamental rhythmic endocrine signal for darkness in the body is controlled by the master clock in the SCN and mainly by the *Period* gene *(Per1)* that has been shown to cycle rhythmically in the pineal gland [74]. For instance, McHill et al. [75] found that, on average, obese individuals consumed most of their calories an hour closer to melatonin onset (biological marker of impending sleep onset) compared to lean individuals.

Also different physiological functions exhibit circadian rhythm: for example glucose tolerance changes during the day showing a poorer glycaemic control in the evening and at night in healthy adults. These changes are influenced by diurnal rhythms in β-cell responsiveness, insulin clearance, and peripheral insulin sensitivity, whilst hepatic insulin sensitivity seems to be less important. However, the circadian rhythm and the inner clock mechanism could be affected by different factors such as light exposure, sleep/wake, physical activity, and food intake. Actually, meal timing is one of the main factors that might influence these physiological functions and, therefore, various health outcomes and body weight control [76]. Meal timing influences either the central master clock (SCN) or peripheral cellular clocks, including Bmal1, Clock, Per1/2, Cry1/2, Rev-erbα/β, Rorα/β, Dbp, Dec1/2, CK1ε/δ, and NPAS2 [74,77].

It is important to underline that peripheral tissues show proper circadian rhythms and cellular clocks. Central and peripheral clocks work together and they are also influenced by food availability. Indeed, regular feeding patterns may synchronize human peripheral clocks and delayed meals could instead influence plasma glucose rhythm but not insulin rhythm [78].

Many genes whose expression is not cyclic may start to follow a circadian rhythm under the pressure of nutritional challenge that modulates PPARs (besides their circadian rhythm) activating many genes by cyclic chromatin recruiting.

**Figure 1:**
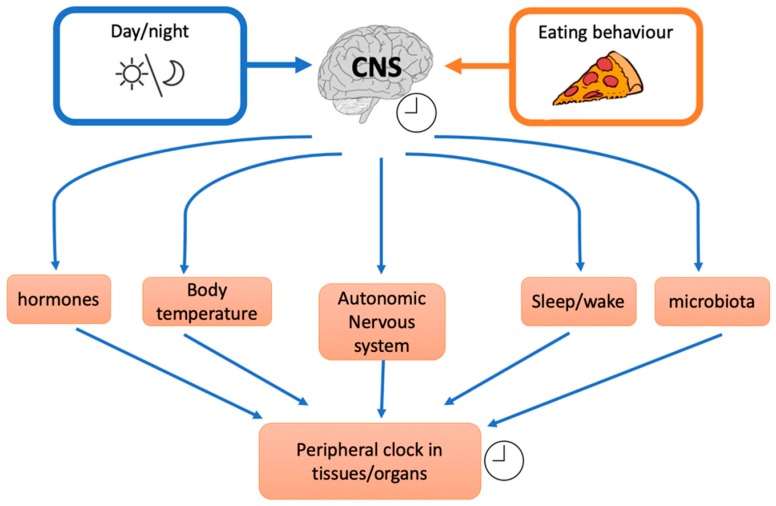
Effects of external factors on the inner central clock that influence different downstream mechanisms and peripheral clocks (CNS: central nervous system).

Even though the mechanisms underlying the effects of meal timing on health outcomes remain obscure, some hypotheses (Figure 1) can tentatively be presented:

(1) Food timing that is out of sync with light/dark cues could induce higher caloric intake due to impaired satiety mechanisms through leptin and ghrelin [79]. Even other hormones involved in metabolism control are affected by circadian misalignment as thyroid hormones [80].
(2) Alteration of gene expression in genes that are associated with evening eating preference and weight loss resistance e.g.,SIRT1, CLOCK 3111T/C, and Perilipin1 [81,82]
(3) Modification of resting energy expenditure: feeding time may affect energy expenditure/basal thermogenesis as core body temperature is controlled by circadian clocks. For example, Rev-erbα is a cellular circadian clock that controls the rhythmic expression of uncoupling protein 1 (UcP1), a fundamental factor for brown adipose tissue thermogenesis [83].
(4) Differences throughout the day in diet-induced thermogenesis (DIT): DIT decreases from morning to night [35,36,84], and some have suggested that “Such circadian thermogenesis could reasonably explain increases in the body mass of persons who skip breakfast” [85].
(5) Circadian clocks influence also insulin resistance through glucose absorption, muscle, fat tissue, and liver insulin sensitivity [86] and food intake or nutritional challenge influence, in turn, circadian clock. Indeed shift workers, transcontinental travelers and people with irregular work schedules often show gastrointestinal symptoms as alterations in bowel habits, constipation, and diarrhoea. These examples indicate that some intestinal functions are rhythmically regulated and that their disruptions lead to health disorders. It was demonstrated that Clock (a peripheral cellular clock) regulates nutrient absorption through the expression of many nutrient transport proteins in the intestine e.g., GLUT2, GLUT5, and Pept1 (a major protein involved in the transport of small peptides from the intestinal lumen to intestinal epithelial cells). However, other external factors could influence the internal clock. For example, NAD^+^ (nicotinamide adenine dinucleotide) levels are influenced by nutritional status and/or physical activity. NAD^+^ influences the SIRT1-dependent deacetylase that activates, through deacetylation, the clock genes BMAL1 (brain-muscle-arnt-Like-protein 1) and PER2 (Period gene 2). Nicotinamide phosphoribosyltransferase (NAMPT) a downstream of BMAL1, has an oscillatory behaviour, therefore modulating the intracellular concentration of NAD+. Thus, in a feedback loop, NAD^+^ concentration regulates SIRT1 that modulates nuclear factors such as PPARγ (peroxisome proliferator-activated receptor gamma) and cofactors as PGC-1α (peroxisome proliferator-activated receptor gamma coactivator 1-alpha) with many effects on different tissues e.g., on hepatic glucose homeostasis (PGC-1α) or adipose tissue lipid mobilization (PPARγ). In general, a regular availability of food (regular meal timing) influences the release, from the gut, of different signals. It has been suggested that signals coming from intestine inform the dorsomedial hypothalamus (DMH) about food availability. Thus, DMH might influence other tissue and regulate food anticipation, digestion, and absorption. Thus, even though circadian genes expressed by gut play an important role, there is some evidence that food, per se, is an important regulator of food entrainment through Clock activity.

## 4. Reducing Meal Frequency: The Case for Time Restricted Feeding

### The Importance of Fasting: What’s New?

If the potential health-promoting effects of less frequent eating are considered sufficient for implementation of this dietary strategy, is consuming one daily meal equivalent to the consumption of two daily meals? In this case, the answer is not merely “less is better”: reducing food intake to only one meal per day may worsen the positive effect of lower meal frequency [87,88]. Therefore, the intake of two (or three) meals per day is perhaps the best option, and the difference between two or three could depend on the length of the daily fasting period they produce.

Much research in recent years suggests a positive health effect of a wide temporal fasting window during the day, i.e., limiting daily food intake to a ~6–8 h time window seems to induce, in humans, many health benefits compared to the normal daily meal distribution (i.e.,three to five meals, spread from breakfast to late dinner), even in isocaloric conditions [89]. It is clear that fasting, in general, exerts many positive effects on health [90], with some features in common with the caloric restriction (CR) approach (protects against diabetes, cancers, heart disease, and neurodegeneration; reduces obesity, hypertension, asthma, and rheumatoid arthritis).

During a typical CR protocol, the daily energy intake is chronically reduced by 20–40%, but meal frequency is maintained. It is well known that CR is a viable tool for health improvement: both animal studies [91] and human research [92,93] showed that this approach could improve many health-related variables.

However, we have to consider the experimental setting of the ab libitum diet and the CR condition to which it is compared in animal experiments. Often, in animal models, the CR condition influences fasting duration. In these experiments, animals in the ad lib diet have unrestricted access to food, not only in quantity but also in frequency, whilst the CR group can only eat within a specific window, usually determined by the researcher’s schedule. In these settings, meals are often spaced out, creating prolonged fasting windows that could influence the outcomes [94]. This is an important issue because fasting is a different approach than traditional CR. We consider fasting as an abstention from food and caloric beverages for a specific interval of time, usually longer than the normal 8 h of sleep. Alternatively, starvation refers to extreme forms of fasting, which result in nutrient deficiencies and other chronic health problems related to the absence of appropriate nutrient intake. Starvation is, actually, a dysregulated condition that leads to a pathological loss of homeostasis related to the reduction in fundamental organ and tissues performance [95]. When considering the different types of fasting programs, we can divide them into two main categories: long-term fasting (LTF) that induces ketosis, and short-term fasting (STF) that does not lead to ketosis. LTF, i.e., fasting with accompanying ketosis, is performed for approximately three days or more. After this period, glucose reserves become depleted and glycogen stores are no longer sufficient to either aid in normal fat oxidation (via oxaloacetate in the Krebs cycle) or to supply energy to the brain and central nervous system (CNS) [96]. Thus, an alternative energy source is needed to maintain the metabolism of the brain. This energy is supplied by the ketone bodies (KBs) acetoacetate (AcAc), 3-hydroxybutyrate (3HB), and acetone, which are generated from acetyl-CoA via a process called ketogenesis, which occurs mainly in the mitochondrial matrix of hepatocytes [96,97]. Ketosis exerts many positive effects on metabolism and numerous cellular pathways, such as increasing stress resistance, lipolysis, mitochondria efficiency, and autophagy (e.g., one of the ketone bodies, b-hydroxybutyrate (D-bHB), is a natural inhibitor of class I and IIa histone deacetylases that repress transcription of the FOXO3a -forkhead box O3 - gene). Moreover, ketone body metabolism reduces the ROS (reactive oxygen species) toxicity through the NADPH system [98]. However, in the context of meal timing and frequency, we want to emphasize the role of STF, which utilizes fasts of insufficient duration to induce ketosis unless used in conjunction with a ketogenic diet. There are several types of STF programs [99]: intermittent fasting (IF) performed as alternate day fasting (ADF) or whole-day fasting for 1–2 days per week, periodic fasting (PF) lasting three or more days every 2–3 weeks, and TRF (Time restricted feeding) whichallows subjects to consume ad libitum energy intake within a defined window of time (from 3–4 h to 10–12 h) [100,101], resulting in a fasting window of 12–21 h per day. For our purposes, we will discuss the TRF because if the number of meals is reduced to two (i.e., breakfast and lunch), and the last meal is consumed between 14:00 h and 16:00 h, this leads to a 12 to 16 h of fasting per 24-h period. It is also worth noting that a substantial amount of research has been conducted during the month-long period of Ramadan fasting observed by practicing Muslims [102]. Ramadan fasting can be considered a form of TRF since food intake is disallowed when it is light outside. However, some notable factors make it difficult to appropriately compare Ramadan fasting to other forms of TRF: the light/dark cycle of eating and fasting is reversed as compared to natural circadian rhythms, the length of fasting window varies based on geographical location and year (Ramadan is set according to the lunar calendar), and different implementations of Ramadan fasting exist (i.e., some eat before the sun rises and after the sun sets, while others only eat after the sun sets). Finally, nearly all studies are observational and last only 4 weeks since this is the duration of Ramadan fasting.

Despite the fact that the duration of fasting during Ramadan (about 16 h) would not typically result in ketosis, it is sufficient to stimulate many of the pathway linked to long term fasting approach, e.g., autophagy [103]. Autophagy, an intracellular process that mediates protein degradation, organelle turnover, and recycling of cytoplasmic components, is a fundamental process to combat cellular stress and preserve normal cell function. In heart and blood vessels, specifically, autophagy plays a fundamental role not only during cardiac embryonic development but also for a normal cardiovascular function. It has been suggested that many of peptides and hormones involved in cardiovascular system physiology are also regulated by autophagy, thus *“it is possible to speculate that dysregulation of autophagy could be associated with hypertension, obesity, diabetes mellitus, and end organ damage”* [104]. As fasting stimulates autophagy, it is likely that these two factors are both related to the demonstrated cardioprotective effect. Indeed, Godar et al. 2015 [105] demonstrated that ADF protects mice from in-vivo ischemia-reperfusion injury, but only in wild-type animals. In mice with impaired autophagy (heterozygous null for Lamp2 coding for lysosomal-associated membrane protein 2), there was not a protective effect, but rather a worsening effect. Another study performed on rats showed that ADF has a cardioprotective effect reducing cerebral infarct size and infarct expansion in a rat model of myocardial infarction (MI) [106].

Fasting affects substrate metabolism, the cardiovascular system and inflammation, as well as exerting potentially powerful effects on circadian rhythms.Increasing the fasting window stimulates fat metabolism and the metabolic switch between glucose oxidation and fat oxidation. Indeed, at between 12 and 36 h of fasting there is an increase of TG (triacylglycerol) lipolysis highlighted by the increase of plasma FFA and glycerol [107]. The metabolic switch typically occurs in the third phase of fasting when glycogen stores in hepatocytes are depleted and the accelerated adipose tissue lipolysis produces an increase in plasma fatty acids and glycerol (21). The fasting period associated with IF and TRF seems to have various positive effects on the cardiovascular system as well: they enhance parasympathetic activity (mediated by the neurotransmitter acetylcholine) in the autonomic neurons that innervate the heart and arteries, resulting in a reduced heart rate and blood pressure [90,108]. Furthermore, TRF could also act on inflammation levels. It is well known that inflammation is related to CHD and atherosclerosis. We demonstrated in humans [100] that an isocaloric TRF approach may reduce many markers of inflammation such as tumour necrosis factor a, interleukin 6, and interleukin 1b, and, at the same time, may increase adiponectin (an anti-inflammatory cytokine). As demonstrated for late eating, fasting also seems to be involved in circadian rhythm regulation or dysregulation. It has been demonstrated that TRF could protect mice against obesity, hyperinsulinemia, hepatic steatosis, and inflammation when fed with a high-fat diet (HFD). The ad libitum HFD rodents also showed altered circadian rhythmicity compared to the TRF rodents. Moreover, TRF improved CREB (cAMP response element-binding protein), mTOR (mechanistic target of rapamycin), and AMP-activated protein kinase (AMPK) pathway function and oscillations of the circadian clock, as well as improving motor coordination [109]. These results could be explained through the considerable crosstalk and the tight interaction between the cellular clocks and the signalling induced by fed/fasted state. For example, we know that fasting, similar to a ketogenic diet, induces the phosphorylation of AMPK, a fundamental actor in mitochondrial biogenesis and function. On the other hand, the fed state stimulates the mechanistic target of rapamycin pathway (mTOR), which promotes anabolic processes during increased energy availability, which could interfere with AMPK pathway. This connection supports the tight relationship between fed/fast state and molecular pathways.

**Figure 2:**
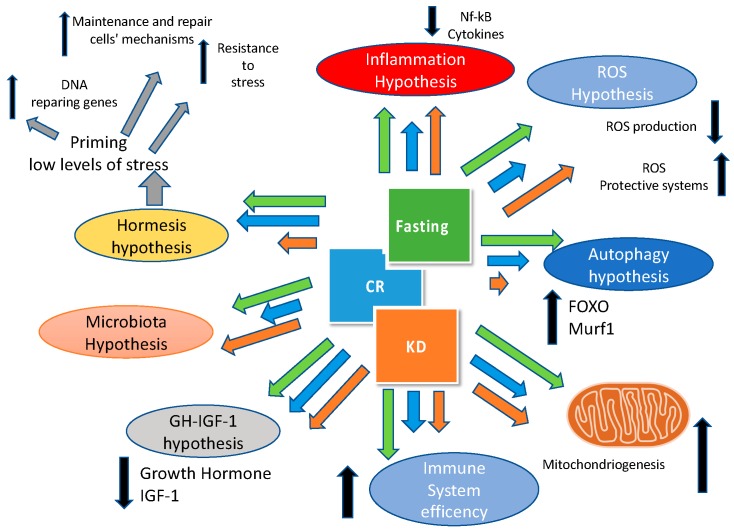
Mechanisms involved in health effects of the ketogenic diet (KD), caloric restriction (CR), and fasting. The size of the arrows is related to the relative effect of KD (orange), CR (blue), and fasting (green) on the different pathways involved (IGF-1: insulin-like growth factor-1; Murf2: Muscle-specific RING finger-2; Nf-kB:nuclear factor kappa-light-chain-enhancer of activated B cells).

Finally, we have to underline that ketogenic diet, caloric restriction and fasting have many pathways and targets in common as shown in Figure 2.

## 5. Meal Frequency and Timing: The Microbiota Connection

We cannot conclude this exploratory review without discussing the role of meal frequency on microbiota. In recent years, this field of research has experienced rapid growth. The collective microbiomal organ provides many fundamental functions such as metabolic, immunological, and infection control. In the last years the gut microbiota has been recognized as an important factor for host general health, immunity, and also energy homeostasis. Changes in microbiota population might cause the development of many metabolic diseases attributable to the modification of the relationship between the bacteria and the host. Up to 100 trillion bacteria constitute the human gut microbiota with 150 times more genes (the microbiome) than the human genome. Abnormalities in gut microbiota composition might have many effects on metabolism in adipose tissue, muscle and liver. Moreover, the gut microbiota has been associated to many metabolic diseases such as obesity, diabetes, chronic low-grade inflammation and, last but not least, cardiovascular disease [110].

Indeed, it has been demonstrated that the composition of microbiota might be a risk factor for CVD. Mice studies have demonstrated the link between gut microbiota dysbiosis and the development of hypertension and vascular dysfunction [111] while in human demonstrated a relationship between negative changes in gut microbiota and primary hypertension has been demonstrated [112]. Gut microbiota converts choline (derived from dietary phosphatidylcholine) to trimethylamine (TMA). In the liver, TMA is converted in trimethylamineN-oxide (TMAO) that promotes atherosclerosis and increases thrombosis risk through the agonist-induced platelet activation [113,114]. In conclusion, available data strongly support the critical role of gut microbiota as a regulatory element in many CVD risk factors.

The microbiota exerts also many actions on the central nervous system, so many that it has been coined the “gut–brain axis.” Diet composition (e.g., fat and fibre content) influences gut microbiota. No data are available in humans concerning meal frequency, whilst some preliminary information about food timing and microbiota are available. Changes in gut microbiota may be stimulated by changes of diurnal feeding and sating rhythms, and it is known that a desynchronization of the suprachiasmatic nucleus, the master clock of the brain, together with a parallel desynchronization of the tissue circadian clocks in skeletal muscle, fat and liver may influence the risk of chronic and metabolic diseases [115]. There is a multifaceted relationship between microbiota and food timing: first, intestinal epithelia cells’ internal circadian clock influences daily glucocorticoid production under the control of the pituitary-adrenal axis, and this rhythm is influenced by microbiota status; second, an alteration of microbiota could lead to a disrupted corticosteroid circadian rhythm influencing food uptake. Moreover, microbiota composition has its variability during the day that could be disrupted by a variety of conditions, for example, jet-lag [116] or high-fat diets. Not only can diet composition exert negative effects on microbiota, but meal timing can also: consuming food outside the normal feeding phase (eating during light time for rodents and during late night in humans) may disturb normal peripheral and central clocks [115]. This desynchronization of internal clocks, and thus the modification of microbiota, is associated with increased risks of metabolic and cardiovascular diseases. Recently it has been demonstrated that a chronic circadian misalignment in mice and a time shift jet–lag in humans induces a dysbiosis; this dysbiosis has been demonstrated to be able to promotes glucose intolerance and obesity in a germ-free mice throughfaecal transplantation [117]. On the other hand, maintaining a correct eating phase (diurnal for humans) and increasing the fasting period (i.e., reducing meal frequency) could positively affect the gut microbiome, reducing gut permeability and improving systemic inflammation. Finally, further studies are needed to explore properly the connection between microbiota and meal frequency and timing.

## 6.Concluding Thoughts

In order to gain a comprehensive picture of the physiological and health effects of meal timing and frequency, multiple lines of research must be integrated and an exploratory review seems to be, in our opinion, the appropriate approach in order to understand, at a glance, the influence of fasting, meal frequency, and timing on cardiovascular diseases. In addition to considering existing evidence of meal frequency and timing per se, research on breakfast consumption, night-time eating, caloric restriction and intermittent fasting can help provide much more awareness about the effects meals manipulation on health outcomes. While a recent meta-analysis reported that high versus low meal frequencies result in negligible differences in body weight and composition changes [117], many of the experimental trials of meal frequency have not adequately considered some of the determinants highlighted in this article which could influence these outcomes (i.e., duration of daily fasting periods and different spacing of meals within the same meal frequency, influence of eating styles on food choices and macronutrient intake, etc.). Additionally, beyond body weight and composition, it is likely that different eating patterns may exert some degree of differential effects on physiological processes, even in isocaloric conditions (Figure 2). Furthermore, the existence of different chronotypes should be taken into account: being larks and owls [118,119], or morning types (M-types) and evening types (E-types), might probably influence also eating behaviour and food metabolism. Even though this classification is not new [120] only in recent years has the association between chronotypes and eating behaviour been investigated. A recent paper by Maukonen and colleagues analysed the associations between chronotype and intakes of energy and macronutrients in the morning and the evening in 1854 participants from the National FINRISK 2007 and FINDIET 2007 studies. They found that, in the morning, E-types showed lower total energy and lower macronutrient intakes except for sucrose (increased intake) compared to M-types.In the evening, E-types had higher intakes of energy, fats, and sucrose than M-types. These data suggest that even chronotype might influence meal patterns [121]

**Figure 3:**
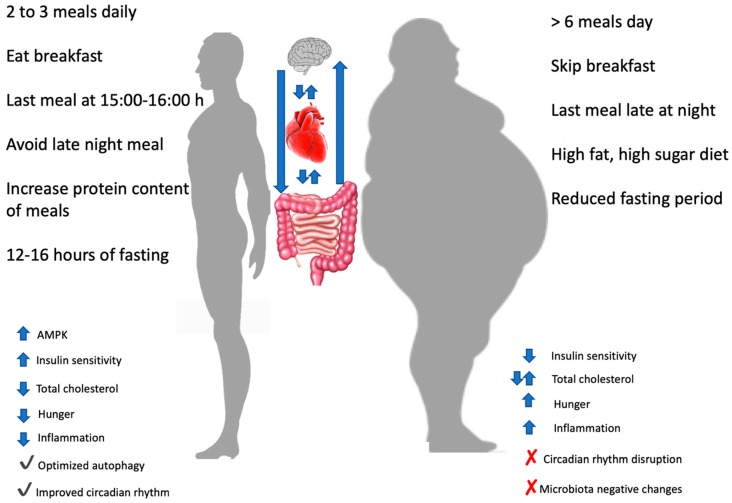
Effects of different meals timing and frequency on different variables. At the centre of the picture the reciprocal influences of brain, heart and gut was showed. AMPK: AMP-activated protein kinase.

Based on the evidence presented in this review, several interesting health-promoting recommendations can be shared with the audience. There may be physiological benefits to consuming a greater proportion of calories earlier in the day, which often involves breakfast consumption, as compared to consuming a large number of calories later at night. There may also be benefits to extending the daily fasting period beyond a standard overnight fast or implementing occasional fasting periods. In order to reconcile these two strategies, an individual could eat from breakfast until mid- to late-afternoon each day (Figure 3). However, it should be considered that this style of eating may not be desirable or feasible for many individuals, as it represents a paradigm shift from traditional eating patterns in many parts of the world.

Additionally, due to the increased access to food associated with evening leisure time, compliance with this recommendation may not be realistic for some. In those cases, it may be beneficial to implement one of the health-promoting strategies (i.e., shift the consumption of most calories earlier in the day or implement a fasting window longer than an overnight fast). The lifestyle approach should include physical activity. Unfortunately, whilst there are few papers on physical exercise and internal clock [122], no data are available about the reciprocal influence of meal time and frequency and physical exercise in humans. This topic is worthy of further investigation.

**Figure 4:**
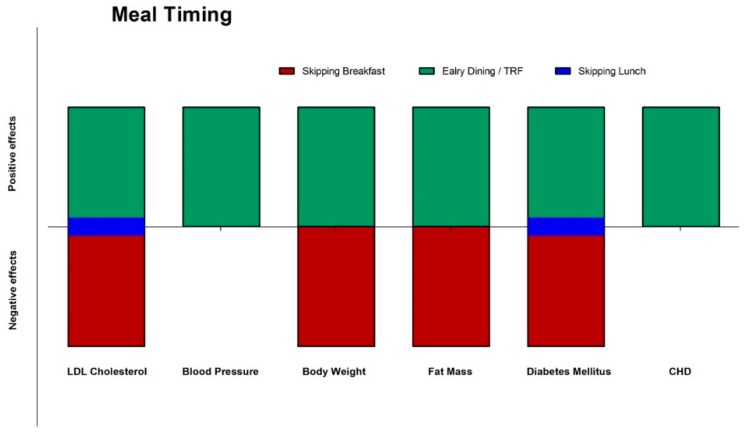
Effects (green: positive; red: negative; blue: neutral) of meal timing on different CVD risks factors and diseases. CHD: coronary heart disease; CVD: cardiovascular disease; TRF: time restricted feeding.

**Figure 5:**
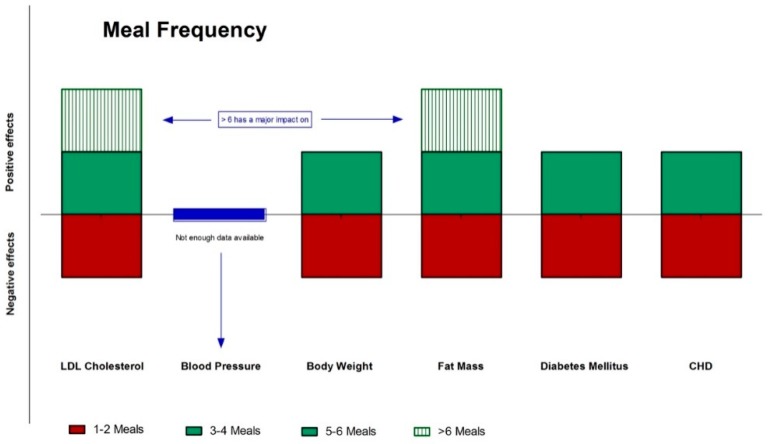
Effects (green: positive; red: negative; blue: neutral) of meal frequency on different CVD risks factors and diseases.CHD: coronary heart disease; CVD: cardiovascular disease.

While a complete picture of the impact of meal timing and frequency in various populations remains to be elucidated, it is likely that manipulation of these variables may be useful in improving health in the human population (Figure 4 and Figure 5). The scientific literature provides sufficient data to suggest that there is a substantial influence of fasting, meal frequency, and timing on health outcomes. These findings underline that not only the food quality but also frequency and timing are crucial for optimal health.
